# Rerefinement of the crystal structure of SnTe_0.73(2)_Se_0.27(2)_ from single-crystal X-ray diffraction data

**DOI:** 10.1107/S2414314622007295

**Published:** 2022-07-19

**Authors:** Silvana Moris, Antonio Galdámez

**Affiliations:** a Centro de Investigación de Estudios Avanzados del Maule (CIEAM), Vicerrectoría de Investigación y Postgrado, Universidad Católica del Maule, Avenida San Miguel 3605, Talca 3480112, Chile; b Universidad de Chile, Facultad de Ciencias, Departamento de Química, Casilla 653, Santiago, Chile; Vienna University of Technology, Austria

**Keywords:** single-crystal, solid solution, rerefinement, tin chalcogenide

## Abstract

Compounds of the solid solution series SnTe_1–*x*_Se_*x*_, derived from pristine SnSe and SnTe, are considered as thermoelectric lead-free materials. The crystal structure re-refinement of NaCl-type SnTe_0.73 (2)_Se_0.27 (2)_ is based on single-crystal X-ray diffraction data and results in higher precision of the bond length [Sn—(Te,Se) = 3.0798 (3) Å] compared to a previous report on basis of powder X-ray data [Krebs & Langner (1964 ▸). *Z. Anorg. Allg. Chem.* **334**, 37–49].

## Abstract 2

The solid solution with composition SnTe_0.73 (2)_Se_0.27 (2)_ crystallizes in the NaCl structure type.

## Graphical abstract

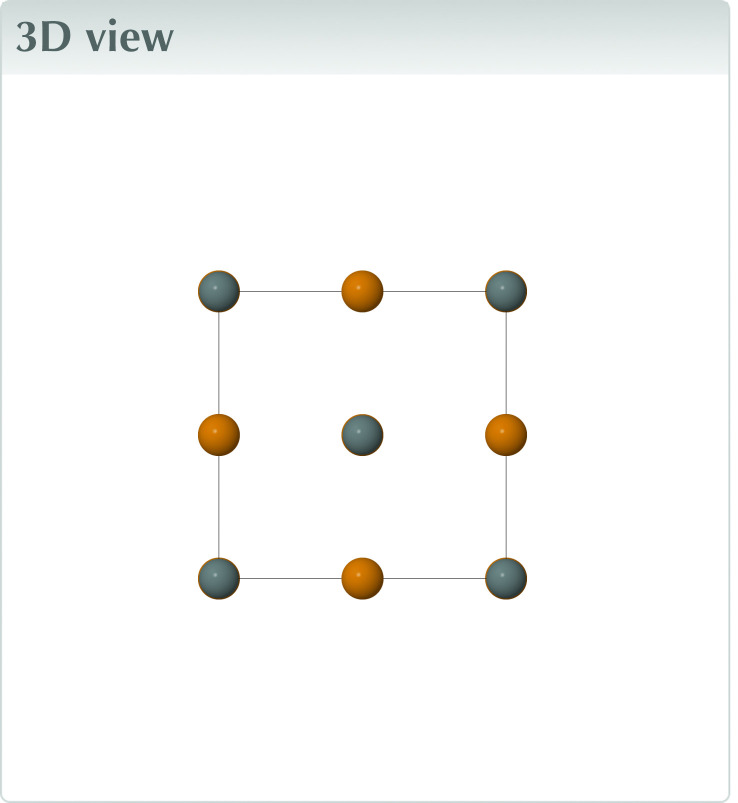

## Structure description

Lead chalcogenides have proven to exhibit an excellent performance as thermoelectric materials. However, due to the current environmental regulations, generating lead-free materials with thermoelectric properties becomes necessary. In this regard, semiconductors of the solid-solution series SnTe_1–*x*_Se_*x*_ have the potential to be good lead-free thermoelectric materials at mid/high temperatures (Banik & Biswas, 2014 ▸). Characterization of phases in the SnTe_1–*x*_Se_*x*_ system on the basis of powder X-ray diffraction data has been reported previously (Krebs & Langner, 1964 ▸; Totani *et al.*, 1968 ▸; Liu & Chang, 1992 ▸; Ariponnammacl *et al.*, 1996 ▸; Majid & Legendre, 1998 ▸; Banik & Biswas, 2014 ▸). The powder patterns were indexed in the cubic crystal system, revealing an NaCl-type crystal structure. The reported unit-cell parameters (Liu & Chang, 1992 ▸) of SnTe_0.9_Se_0.1_ and SnTe_0.75_Se_0.25_ are *a* = 6.2433 Å and *a* = 6.2188 Å, respectively. The SnTe_1–*x*_Se_*x*_ (*x* = 0 to 0.15) samples could be viewed as solid solutions that obey Vegard’s law (Banik & Biswas, 2014 ▸). The parameter *a* of the cubic unit cell increases from approximately 6.23 to 6.27 Å with decreasing Se content *x*, as determined from the powder pattern.

**Figure 1:**
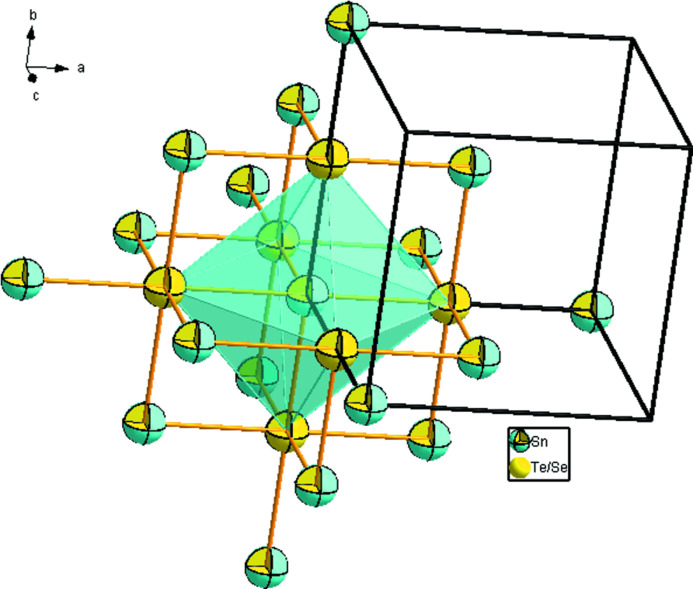
A view of the NaCl-type crystal structure of SnTe_0.73 (2)_Se_0.27 (2)_. Displacement ellipsoids are drawn at the 50% probability level.

Here, we report the crystal structure rerefinement of the solid solution with composition SnTe_0.73 (2)_Se_0.27 (2)_ from single-crystal X-ray diffraction data. The crystal structure of SnTe_0.73 (2)_Se_0.27 (2)_ likewise adopts the NaCl type (Fig. 1 ▸) with an inter­mediate value of *a* [6.1595 (5) Å] between those of the binary compounds SnTe (6.314 Å) and SnSe (5.99 Å), which is attributed to the different radii of Te and Se. The cell parameter reported by Liu & Chang (1992 ▸) of *a* = 6.2188 Å for SnTe_0.75_Se_0.25_ at room temperature is somewhat higher than that of SnTe_0.73 (2)_Se_0.27 (2)_ determined at 150 K. The present study allowed for a higher precision with respect to the bond lengths in SnTe_0.73 (2)_Se_0.27 (2)_, which is Sn—(Te,Se) = 3.0798 (3) Å.

## Synthesis and crystallization

Single crystals of the title compound were obtained seren­dipi­tously by application of the high-temperature ceramic method. Powders of silver (99.99%), tin (99.99%), bis­muth (99.999%), selenium (99%) and tellurium (99%) were weighted in an molar ratio of 1:2:1:2.5:2.5. All manipulations were carried out under an argon atmosphere. The reaction mixture was sealed in an evacuated silica ampoule and placed in a programmable furnace (Figueroa-Millon *et al.*, 2018 ▸). The ampoule was slowly heated from room temperature to 1023 K at a rate of 333 K min^−1^ to the maximum temperature and held for 7 d, followed by slow cooling to room temperature at a rate of 278 K h^−1^. The reaction product consisted of a gray metallic powder (yield ∼99%) accompanied by black octa­hedrally shaped single crystals (yield ∼1%) that were manually separated for the X-ray diffraction study. The refined composition of the measured crystal is SnTe_0.73 (2)_Se_0.27 (2)_.

## Refinement

Crystal data, data collection, and structure refinement details are summarized in Table 1 ▸. For the shared chalcogenide site, the sum of site occupation factors (SOF) was constrained to 1, and the anisotropic displacement parameters were constrained to be the same. The crystal under investigation consisted of two domains with approximately equal contribution to the intensity data. The integration procedure showed that the reflections of each domain were clearly separated. For the final intensity data only one domain was used.

## Supplementary Material

Crystal structure: contains datablock(s) 1R, I. DOI: 10.1107/S2414314622007295/wm4169sup1.cif

Structure factors: contains datablock(s) I. DOI: 10.1107/S2414314622007295/wm4169Isup2.hkl

CCDC reference: 2190397

Additional supporting information:  crystallographic information; 3D view; checkCIF report

## Figures and Tables

**Table 1:** Experimental details

Crystal data	
Chemical formula	SnTe_0.73_Se_0.27_
*M*_r_	233.28
Crystal system, space group	Cubic, *F* *m* 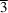 *m*
Temperature (K)	150
*a* (Å)	6.1595 (5)
*V* (Å^3^)	233.69 (6)
*Z*	4
Radiation type	Mo *K*α
μ (mm^−1^)	23.61
Crystal size (mm)	0.10 × 0.08 × 0.07

Data collection	
Diffractometer	Nonius KappaCCD
Absorption correction	Multi-scan (*SADABS*; Krause *et al.*, 2015 ▸)
*T*_min_, *T*_max_	0.103, 0.205
No. of measured, independent and observed [*I* > 2σ(*I*)] reflections	396, 33, 33
*R*_int_	0.042
(sin θ/λ)_max_ (Å^−1^)	0.703

Refinement	
*R*[*F* ^2^ > 2σ(*F* ^2^)], *wR*(*F* ^2^), *S*	0.045, 0.115, 1.49
No. of reflections	33
No. of parameters	4
Δρ_max_, Δρ_min_ (e Å^−3^)	1.92, −1.84

Computer programs: *COLLECT* (Bruker, 1997-2004 ▸), *DIRAX/LSQ* (Duisenberg *et al.*, 2003 ▸), *EVALCCD* (Duisenberg *et al.*, 2003 ▸), *olex2.solve* (Bourhis *et al.*, 2015 ▸), *SHELXL* (Sheldrick, 2015 ▸), *OLEX2* (Dolomanov *et al.*, 2009 ▸) and *publCIF* (Westrip, 2010 ▸).

## full crystallographic data

### Crystal data

**Table:**

SnTe_0.73_Se_0.27_	Mo *K*α radiation, λ = 0.71073 Å
*M**_r_* = 233.28	Cell parameters from 553 reflections
Cubic, *F**m*3*m*	θ = 5.7–39.8°
*a* = 6.1595 (5) Å	µ = 23.61 mm^−^^1^
*V* = 233.69 (6) Å^3^	*T* = 150 K
*Z* = 4	Octahedron, black
*F*(000) = 389	0.10 × 0.08 × 0.07 mm
*D*_x_ = 6.631 Mg m^−^^3^	

### Data collection

**Table:**

Nonius KappaCCD diffractometer	33 independent reflections
Radiation source: Enraf Nonius FR590	33 reflections with *I* > 2σ(*I*)
Graphite monochromator	*R*_int_ = 0.042
Detector resolution: 9 pixels mm^-1^	θ_max_ = 30.0°, θ_min_ = 5.7°
CCD rotation images, thin slices scans	*h* = −8→8
Absorption correction: multi-scan (*SADABS*; Krause *et al.*, 2015)	*k* = −7→8
*T*_min_ = 0.103, *T*_max_ = 0.205	*l* = −6→8
396 measured reflections	

### Refinement

**Table:**

Refinement on *F*^2^	4 parameters
Least-squares matrix: full	0 restraints
*R*[*F*^2^ > 2σ(*F*^2^)] = 0.045	*w* = 1/[σ^2^(*F*_o_^2^) + (0.0583*P*)^2^ + 1.8772*P*] where *P* = (*F*_o_^2^ + 2*F*_c_^2^)/3
*wR*(*F*^2^) = 0.115	(Δ/σ)_max_ < 0.001
*S* = 1.49	Δρ_max_ = 1.92 e Å^−^^3^
33 reflections	Δρ_min_ = −1.84 e Å^−^^3^

### Special details

**Table:**

Geometry. All esds (except the esd in the dihedral angle between two l.s. planes) are estimated using the full covariance matrix. The cell esds are taken into account individually in the estimation of esds in distances, angles and torsion angles; correlations between esds in cell parameters are only used when they are defined by crystal symmetry. An approximate (isotropic) treatment of cell esds is used for estimating esds involving l.s. planes.

### Fractional atomic coordinates and isotropic or equivalent isotropic displacement parameters (Å^2^)

**Table:**

	*x*	*y*	*z*	*U*_iso_*/*U*_eq_	Occ. (<1)
Sn	0.000000	0.000000	0.000000	0.0199 (10)	
Te	0.000000	0.000000	0.500000	0.0330 (12)	0.73 (2)
Se	0.000000	0.000000	0.500000	0.0330 (12)	0.27 (2)

### Atomic displacement parameters (Å^2^)

**Table:**

	*U*^11^	*U*^22^	*U*^33^	*U*^12^	*U*^13^	*U*^23^
Sn	0.0199 (10)	0.0199 (10)	0.0199 (10)	0.000	0.000	0.000
Te	0.0330 (12)	0.0330 (12)	0.0330 (12)	0.000	0.000	0.000
Se	0.0330 (12)	0.0330 (12)	0.0330 (12)	0.000	0.000	0.000

### Geometric parameters (Å, º)

**Table:**

Sn—Te^i^	3.0798 (3)	Sn—Te^iii^	3.0798 (3)
Sn—Te	3.0798 (3)	Sn—Te^iv^	3.0798 (3)
Sn—Te^ii^	3.0798 (3)	Sn—Te^v^	3.0798 (3)
			
Te^i^—Sn—Te	90.0	Sn^vi^—Te—Sn	90.0
Te^i^—Sn—Te^ii^	90.0	Sn^vi^—Te—Sn^vii^	90.0
Te—Sn—Te^ii^	180.0	Sn—Te—Sn^vii^	180.0
Te^i^—Sn—Te^iii^	90.0	Sn^vi^—Te—Sn^viii^	90.0
Te—Sn—Te^iii^	90.0	Sn—Te—Sn^viii^	90.0
Te^ii^—Sn—Te^iii^	90.0	Sn^vii^—Te—Sn^viii^	90.0
Te^i^—Sn—Te^iv^	90.0	Sn^vi^—Te—Sn^ix^	90.0
Te—Sn—Te^iv^	90.0	Sn—Te—Sn^ix^	90.0
Te^ii^—Sn—Te^iv^	90.0	Sn^vii^—Te—Sn^ix^	90.0
Te^iii^—Sn—Te^iv^	180.0	Sn^viii^—Te—Sn^ix^	180.0
Te^i^—Sn—Te^v^	180.0	Sn^vi^—Te—Sn^x^	180.0
Te—Sn—Te^v^	90.0	Sn—Te—Sn^x^	90.0
Te^ii^—Sn—Te^v^	90.0	Sn^vii^—Te—Sn^x^	90.0
Te^iii^—Sn—Te^v^	90.0	Sn^viii^—Te—Sn^x^	90.0
Te^iv^—Sn—Te^v^	90.0	Sn^ix^—Te—Sn^x^	90.0

Symmetry codes: (i) *x*+1/2, *y*, *z*−1/2; (ii) *x*, *y*, *z*−1; (iii) *x*, *y*−1/2, *z*−1/2; (iv) *x*, *y*+1/2, *z*−1/2; (v) *x*−1/2, *y*, *z*−1/2; (vi) *x*+1/2, *y*, *z*+1/2; (vii) *x*, *y*, *z*+1; (viii) *x*, *y*−1/2, *z*+1/2; (ix) *x*, *y*+1/2, *z*+1/2; (x) *x*−1/2, *y*, *z*+1/2.
